# A Mobile App (Tpro) for Symptom Management in Patients With Deep Vein Thrombosis Based on Patient-Reported Outcomes: Design and Development Using an Iterative Convergent Mixed Methods Approach

**DOI:** 10.2196/92738

**Published:** 2026-07-03

**Authors:** Qiaodan Lu, Yafei Liu, Manna Shao, Lei Wang

**Affiliations:** 1 Department of Vascular Surgery, Chinese Academy of Medical Sciences and Peking Union Medical College, Peking Union Medical College Hospital, No. 1, Shuaifuyuan, Dongcheng District, Beijing, 100730, China, 86 13910016630; 2 Department of Nursing, Chinese Academy of Medical Sciences and Peking Union Medical College, Peking Union Medical College Hospital, Beijing, China

**Keywords:** deep vein thrombosis, patient-reported outcomes, mobile app, mixed methods research, iterative design

## Abstract

**Background:**

Deep vein thrombosis (DVT) is a significant global health issue, often associated with a high symptom burden and reduced quality of life, especially after discharge. Traditional symptom management models are typically passive, clinician-centered, and lack real-time monitoring and feedback, which can lead to delayed interventions and poor patient engagement. While mobile health (mHealth) interventions offer a promising alternative, they require rigorous usability testing to ensure both efficacy and adoption.

**Objective:**

This study aimed to design and develop Tpro (developed by the Department of Vascular Surgery, Peking Union Medical College Hospital), a patient-reported outcome (PRO)–based mobile app for patients with DVT. Using an iterative convergent mixed methods design, the app seeks to enable proactive symptom monitoring, health education, clinician-patient interaction, and peer support, thereby optimizing its usability, functionality, and alignment with patient needs.

**Methods:**

The development followed an iterative convergent mixed methods design, comprising predevelopment and iterative optimization phases. Initial functions were informed by qualitative interviews with 14 patients with DVT. Over 4 iterative cycles, qualitative feedback and quantitative usability data (including task completion rates and User Interface Usability Questionnaire [UIUQ] scores) were concurrently collected, analyzed, and integrated via joint displays to guide refinements in content, interface, and system architecture until usability benchmarks were met.

**Results:**

The final Tpro app encompasses 4 core modules, including gamified symptom reporting, multimodal health education, clinician-patient communication, and a peer support community. Iterative testing identified and resolved key usability issues. The final prototype demonstrated high usability, achieving an excellent UIUQ score (mean 89, SD 13) and a task completion rate of 92% (12/13), indicating high user acceptance and operational reliability.

**Conclusions:**

Applying the iterative convergent mixed methods approach enabled the systematic and user-centered development of Tpro. This methodology effectively integrated diverse stakeholder feedback into a functional and engaging patient-reported outcome–based app, ready for subsequent efficacy trials. This approach offers a replicable model for developing digital health tools in complex clinical contexts.

## Introduction

Deep vein thrombosis (DVT) has emerged as a significant contributor to cardiovascular disease-related mortality on a global scale [1]. Individuals afflicted with DVT frequently experience a substantial array of acute and chronic symptoms, including persistent pain, swelling, functional impairment, and psychological distress, leading to a diminished quality of life and subsequently escalating the overall economic burden on health care systems worldwide [2].

Effective symptom management is crucial for predicting adverse prognostic events, preventing complications, optimizing treatment outcomes, improving overall health, and enhancing quality of life in patients with DVT [3]. However, this traditional symptom management model operates on a passive-reactive and clinician-centric framework, predominantly reliant on infrequent routine ward rounds or scheduled in-person hospital consultations [4]. This approach inherently lacks mechanisms for active, real-time monitoring of patients’ symptoms, particularly those occurring between visits or in out-of-hospital settings, and fails to adequately incorporate the patient’s subjective experience and perspective. Consequently, health care providers are often unable to promptly identify worsening symptoms or address emerging patients' needs, leading to significant delays in intervention and a critical disconnect between the patient’s actual clinical status and the health care system’s responsiveness.

The integration of patient-reported outcomes (PRO) into symptom management exemplifies a patient-centered approach, fully aligning with core principles of patient-centered medical care. This model empowers patients to autonomously monitor symptoms, alleviate symptom burden, and enhance quality of life [5]. While widely applied in clinical trials, drug approval, and tumor prognosis, this model has yet to be integrated into symptom management for patients with DVT [6,7].

Mobile health (mHealth) apps offer a promising platform to facilitate PRO collection and support self-management, enabling patients to engage in disease management beyond clinical settings [8]. Nevertheless, many mHealth solutions face poor adoption due to insufficient usability and a lack of end-user involvement during development [9]. Currently, there is no mHealth app based on PRO that has been developed with a rigorous user-centered design approach specifically tailored to address the symptom management needs of patients with DVT.

To tackle these obstacles, this study used an iterative convergent mixed methods design [10], a robust methodology that concurrently gathers and evaluates qualitative and quantitative data throughout various development phases, to create Tpro, an mHealth app centered on PRO specifically for individuals with DVT. The app is structured to facilitate proactive symptom monitoring, deliver health education, enhance clinician-patient communication, and provide peer support. In practice, patients are guided through a daily workflow and after receiving a customizable reminder, they complete a symptom report, during which they can message their clinician. Upon submission, tailored health education content is automatically unlocked. A knowledge library and a peer community remain available for self-directed use at any time. By merging the PRO framework with iterative user-centered development, Tpro strives to enhance usability, functional significance, and alignment with patient requirements, thereby promoting a more adaptive and patient-involved approach to managing DVT symptoms.

## Methods

### Overview of Development Methodology

The development of Tpro used an iterative convergent mixed methods design as formalized by Alwashmi et al [10]. This approach is characterized by the concurrent collection and integration of qualitative and quantitative data across cyclical iterations, continuing until predefined usability endpoints are achieved [10]. In this study, the process was structured across 2 interlinked phases, namely a predevelopment phase, which defined core functions and wireframes based on prior qualitative inquiry and clinical consensus, and an iterative optimization phase consisting of 4 consecutive cycles of prototype testing and refinement. Within each cycle, qualitative feedback and quantitative usability metrics were systematically integrated through joint displays and team interpretation sessions, directly informing real-time design modifications. This evidence-driven, user-centered process continued until design saturation was achieved, that is, until no new critical usability issues emerged and key performance metrics stabilized.

### Predevelopment Phase

#### Defining Core Functions Through Qualitative Inquiry

The primary function of Tpro was derived from qualitative interviews conducted in the initial phase of the study with 14 patients with DVT engaged in 6-month out-of-hospital self-management [11]. The findings suggest that enhancing health education can enhance patients’ understanding, improve doctor-patient communication, facilitate monitoring, and establish a support system to enhance the quality of out-of-hospital patient with DVT management [11].

Expanding on these results, the researchers, leveraging their substantial clinical expertise in managing patients with DVT (each investigator with at least 5 years of experience), reached a consensus. As a result, the primary roles of Tpro were identified as symptom monitoring, health education, facilitating medical-patient interaction, and fostering peer support among patients.

#### Wireframing and Content Development

Wireframes, shown as line diagrams and geometric placeholders, define spatial hierarchy, modular component organization, and relationships among interactive elements. They explicitly convey an app’s basic architectural topology and provide a visual framework for design and development teams. Wireframes are used for concept communication, user-flow optimization, and the establishment of the structural foundation during the initial development stage [12]. The researchers generated a wireframe diagram of Tpro based on its 4 primary functions. As shown in Multimedia Appendix 1, the wireframe illustrates the initial layout and user flow among the symptom monitoring, education, communication, and community modules.

Following the definition of the app’s core functions and structure, the app was named “Tpro,” a term derived from combining the target condition (“Thrombosis”) with the core methodology (“Patient-Reported Outcomes”). The multimedia content was subsequently developed through a systematic, evidence-based process. This included original anatomical illustrations (eg, diagrams of human vasculature) created by medical illustrators; educational texts and instructional videos, adapted from existing patient education materials and produced in collaboration with vascular nursing specialists, with all materials undergoing health literacy review [13]; a knowledge question bank clinically validated by experts; and gamified learning scripts, designed based on behavior change theory [14] and integrated with the core text and image materials.

### Iterative Optimization Phase

**Figure 1.**
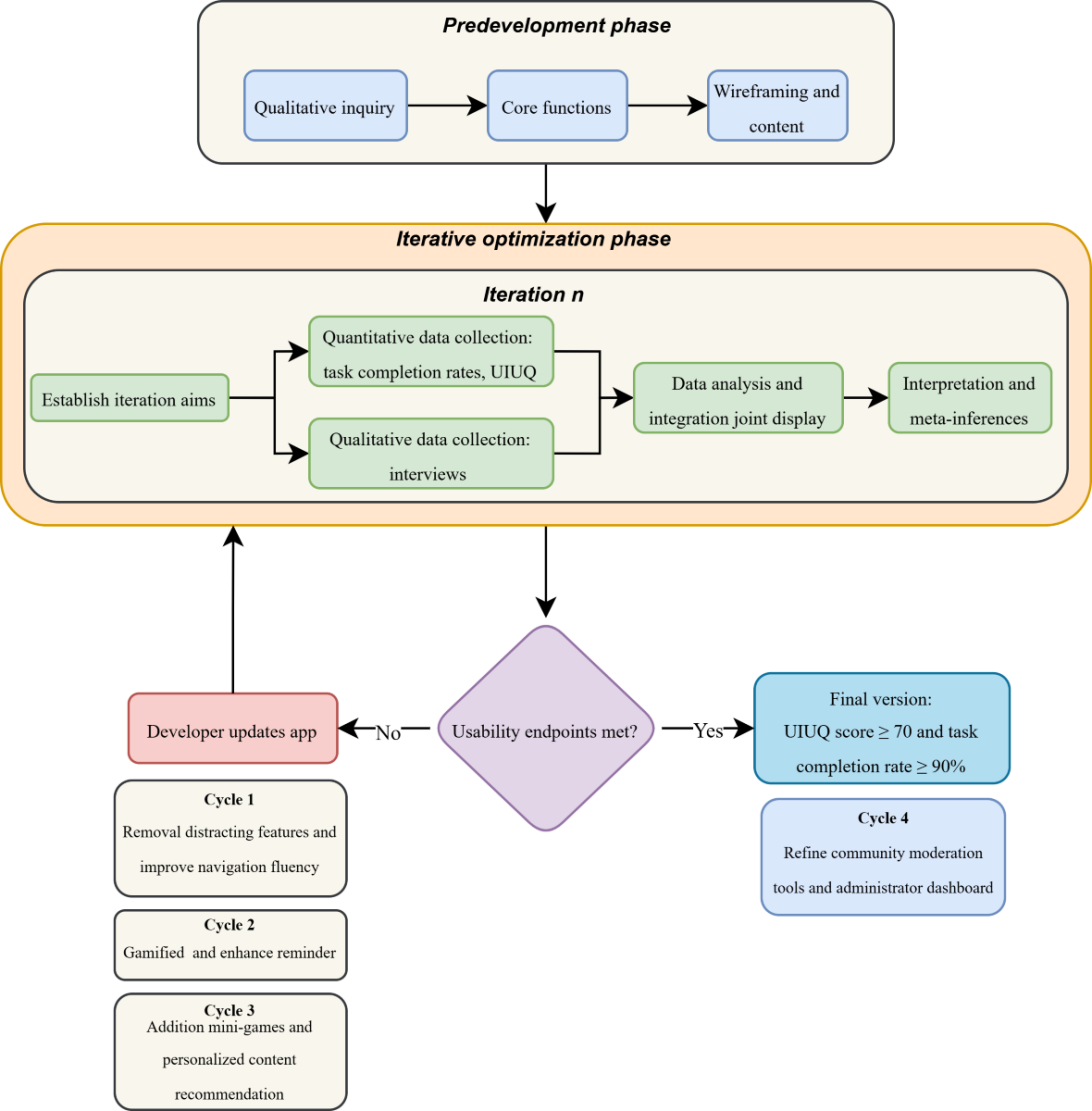
Flowchart of the 4-cycle iterative convergent mixed methods optimization process for Tpro development. UIUQ: User Interface Usability Questionnaire.

We conducted 4 complete iterative cycles of testing and refinement between November 2024 and March 2025. Each cycle followed the structured process outlined in Figure 1 and incorporated the integration procedures detailed below. Representative screenshots of key interface updates from each cycle (Cycle 1-Cycle 4) are provided in Multimedia Appendix 2.

#### Integration in the Research Questions Dimension

The study used 3 types of research questions that guided each iteration:

(1) Mixed methods aim: to illustrate, explore, and measure how to improve the usability and functionality of Tpro for DVT symptom management

(2) Quantitative research questions: focused on measuring effectiveness (task completion rates) and satisfaction (User Interface Usability Questionnaire [UIUQ] scores) [15]. Task completion rates were defined as the proportion of participants who successfully completed both the symptom reporting process and at least one health education activity (knowledge reading or quiz completion) within the Tpro app. The UIUQ developed by Chinese scholar Liu consists of 20 items scored on a 0‐5 scale, yielding a total score of 0‐100 points. Higher scores indicate better interface usability. Based on the total score: below 50 points represents unacceptable usability, 50‐70 points indicates marginally acceptable usability, and above 70 points reflects satisfactory usability.

(3) Qualitative research questions: centered on understanding how and why users interacted with Tpro as they did, their experiences with specific features, and suggestions for improvement. Table 1 summarizes the data collection methods and integration timing used in this study.

**Table 1.** Data collection methods and integration timing. This table summarizes the mixed methods data collection strategy, specifies the types of data collected, and outlines how they were integrated concurrently to inform real-time design decisions during each development cycle.

Data type	Methods	Collection timing	Integration approach
Quantitative	UIUQ^a^, task completion rates	During and immediately after app use	Concurrent with qualitative data collection
Qualitative	Semistructured interviews	During app use and in postuse debriefing	Immediate feedback into quantitative understanding

a UIUQ: User Interface Usability Questionnaire.

#### Integration in the Data Collection Dimension

We implemented 3 integration strategies during data collection: (1) matching: qualitative questions were intentionally matched to quantitative constructs. For example, when low task completion rates were observed quantitatively, we asked, “I noticed you hesitated when completing the task, can you tell me more about what was unclear?” (2) Diffracting: qualitative questions explored aspects beyond quantitative measures, such as emotional responses to the gamified interface, trust in medical recommendations, and preferences for community interaction styles. And (3) expanding: when quantitative and qualitative findings diverged, we expanded questioning to understand both perspectives. For instance, when users reported high satisfaction qualitatively but gave medium UIUQ scores quantitatively, we explored this discrepancy specifically.

#### Collection of Qualitative Data and Researcher Reflexivity

Qualitative data within each cycle were collected primarily via semistructured interviews, conducted by 2 researchers (MS and YL). Both interviewers held clinical research backgrounds and had completed formal training in qualitative interview techniques. They had no prior clinical or therapeutic relationship with the patient participants, nor had they engaged in any discussions with participants regarding the Tpro app or DVT management prior to the study, which was explicitly explained at the outset to minimize social desirability bias and encourage candid feedback. Researchers maintained reflexive journals to document and bracket their own assumptions prior to and following interviews. Data collection proceeded within each cycle until informational saturation was attained on the usability themes specific to that cycle’s prototype, which was determined when subsequent interviews yielded no new insights related to the core evaluation objectives.

#### Integration in the Data Analysis Dimension

We used a combined independent and interactive analysis strategy:

(1) Interactive analysis: during early iterations (Cycles 1‐2), researchers analyzed qualitative and quantitative findings in real-time as data were collected, allowing immediate exploration of emerging issues

(2) Independent intramethod analysis: in later iterations (Cycles 3‐4), qualitative data underwent inductive thematic analysis and quantitative data were analyzed statistically prior to integration. Specifically, for thematic analysis, 2 researchers (QL and LW) independently coded transcripts from initial cycles to develop a preliminary codebook. Discrepancies were resolved through discussion until consensus was reached, thereby enhancing interpretive reliability. The refined codebook was then applied to subsequent cycles, with ongoing peer debriefing within the research team to ensure analytical rigor. Representative quotations illustrating key themes are provided in Multimedia Appendix 3.

(3) Data integration procedures: we used joint displays to visually represent the integration of qualitative and quantitative findings (see Table 2 for example). The fit of the 2 data types was examined for confirmation, complementarity, expansion, or discordance. Discordant findings were resolved through additional data collection or reanalysis.

**Table 2.** Joint display from Cycle 1 showing data integration and resulting design decisions. This table provides a concrete example of the convergent mixed methods analysis, showing how specific usability metrics were interpreted alongside direct user feedback to identify root causes and translate them into actionable design modifications.

Quantitative findings	Qualitative findings	Integration insights	Design decisions
Around 52% (11/21) of users failed to complete symptom reporting or knowledge learning first attempt	“I got distracted by the chat function on main screen and forgot to report symptoms” (Patient 03) “app is super convenient, but I don’t really have time to learn about thrombosis. I just check it out from time to time” (Patient 10) “The knowledge base still has some room for improvement. There are so many topics I want to learn about! It would be great to have more educational mini-games” (Patient 11, Patient 08)	Core functionality was being undermined by secondary features and cognitive load	Removed chat function from main screen to dedicated section Added interactive mini-games and expanded knowledge content Enhanced personalized reminder system based on usage patterns
UIUQ^a^ score for efficiency (86/100)	“I wasn’t sure if my report went through-no confirmation” (Patient 07) “The navigation feels jumpy between sections” (Patient 05)	Lack of feedback and inconsistent navigation patterns created uncertainty and frustration	Added clear submission confirmations Standardized navigation patterns across all modules Improved transition animations

a UIUQ: User Interface Usability Questionnaire.

#### Integration in the Interpretation Dimension

During each iteration cycle, the research team conducted interpretation sessions where quantitative and qualitative findings were merged to draw metainferences that considered both types of data. These sessions followed a structured process:

(1) Data triangulation: comparing quantitative metrics with qualitative themes

(2) Pattern identification: looking for convergent, complementary, or divergent findings

(3) Priority setting: determining which issues to address in the next iteration based on severity and frequency

(4) Solution generation: brainstorming specific design modifications

### Participant Recruitment

Participant recruitment used a combination of convenience and purposive sampling strategies to engage key stakeholder groups. Patient participants were recruited via convenience sampling from the vascular surgery outpatient clinic of Peking Union Medical College Hospital. Inclusion criteria for patients required a confirmed DVT diagnosis, proficiency in smartphone use, and expressed willingness to engage in multiple testing cycles. Exclusion criteria encompassed significant cognitive impairment, visual disabilities preventing effective smartphone interaction, or concurrent participation in other digital health studies. Clinical experts, including vascular surgeons and specialized nurses, were purposively recruited from the Department of Vascular Surgery at Peking Union Medical College Hospital based on their documented clinical expertise and experience in DVT management. Similarly, nonmedical technical evaluators with specialized backgrounds in usability engineering, software development, or human-computer interaction were purposively selected from the collaborating technical team involved in this research project.

### Sample Size Justification

The sample size for iterative usability testing was determined a priori based on established principles of user-centered design and the iterative convergent design framework adopted for this study. Foundational usability research indicates that testing with 5-12 users per iteration is sufficient to identify the majority of interface problems [16]. Aligned with this framework [10], the primary endpoint of the iterative process was not statistical power but the achievement of thematic and design saturation, the point at which additional testing cycles yield no new critical usability themes, barriers, or actionable design feedback. Recruitment and iterative testing continued until this saturation criterion was operationally confirmed, which was assessed through the stabilization of quantitative usability metrics (task completion rate and UIUQ score) in consecutive cycles.

### Ethical Considerations

The study received approval from the Institutional Review Board of Peking Union Medical College Hospital (approval number: I-24PJ1204). Written informed consent was obtained from all participants prior to their enrollment in the study. We attest that the privacy and confidentiality of all research subjects’ data and identities were strictly maintained throughout the study.

### Development Technical Specifications

Tpro is constructed using the Flutter (Google LLC) framework, enabling simultaneous app development for Android, iOS, Web, and desktop (Windows, macOS, and Linux), thereby streamlining code writing across platforms, shortening project timelines, and reducing costs. Embedded pages are built on a Java-based low-code platform. The Tpro app is compatible with Android 5 and higher, as well as iOS 11 and newer versions. Initially developed in Chinese to align with regional preferences, Tpro will be offered in English and other languages in the future.

During the iterative testing cycles, the Tpro package was installed on participants’ personal devices via a secure, authenticated download link, or was made accessible on preconfigured study devices where necessary. Access required individual user accounts verified by the clinical research team. This deployment strategy ensured controlled distribution and facilitated rapid iterative updates during the optimization cycles.

### Data Security and Privacy Protection

The Tpro system was designed with a multilayered security and privacy architecture. All data transmission was protected by TLS encryption, and sensitive data were encrypted at rest. The app backend was deployed on a secure, internal server to ensure physical and administrative data control. Access to patient information was strictly governed by role-based permissions within the clinician admin portal, and all accesses were logged. In the peer community, user identities were anonymized. These measures were designed to comply with relevant data protection regulations and were reviewed as part of the ethical approval process.

## Results

### Participant Characteristics and Iterative Testing Process

The iterative development of Tpro was conducted over 4 cycles spanning a 4-month period, engaging a total of 21 participants. The patient cohort consisted of 12 individuals with confirmed DVT (8 males and 4 females), with an age range of 25-72 years, all recruited from the vascular surgery outpatient clinic. Their educational backgrounds ranged from elementary school to master's degree, and they presented with diverse DVT locations (including iliofemoral, calf, and femoral veins) and varying symptom severities. The multidisciplinary evaluation team comprised 6 clinical experts: 2 vascular surgeons (mean 10.5, SD 6.4 years of specialization) and 4 specialized nurses (mean 6, SD 1.4 years of experience). Additionally, the team included 3 nonmedical technical evaluators specializing in usability research and software engineering (mean 8, SD 3.6 years of relevant experience).

Engagement across the iterative cycles was structured to balance depth of longitudinal feedback with the introduction of fresh perspectives, continuing until thematic and data saturation were achieved. All 6 clinical experts and all 3 technical evaluators participated in all 4 cycles, providing consistent expert and technical oversight. Among the 12 patients, 4 completed all 4 cycles, forming a core longitudinal feedback group, while the remaining 8 patients participated in 2-3 cycles, ensuring that new user perspectives were incorporated at each stage. Consequently, a total of 13 participants (4 patients, 6 clinical experts, and 3 technical evaluators) completed all 4 testing cycles, thereby providing the continuous and in-depth feedback necessary to reach this saturation endpoint. The comprehensive demographic, clinical, professional, and cycle-by-cycle participation details for each individual are provided in Multimedia Appendix 4.

### Evolution of Usability Metrics

The systematic application of the iterative convergent approach yielded substantial improvements across all usability metrics. The UIUQ score demonstrated progression from an initial score of 86 in the first cycle to a final score of 89 in the fourth cycle. Task completion rates showed consistent improvement, rising from 46% (6/13) in initial testing to 92% (12/13) in the final evaluation (Table 3).

**Table 3.** Usability metrics evolution across 4 iterative cycles (n=13). This table presents the progression of key quantitative usability indicators, including the overall User Interface Usability Questionnaire (UIUQ) score and the task completion rate, demonstrating the systematic improvement achieved through the iterative refinement process.

Metric	Cycle 1	Cycle 2	Cycle 3	Cycle 4
Overall UIUQ^a^ score, mean (SD)	86 (6)	87 (6)	88 (8)	89 (13)
Task completion rate, n (%)	6 (46)	10 (77)	11 (85)	12 (92)

a UIUQ: User Interface Usability Questionnaire.

### Key Refinements Through Integrated Data Analysis

The integrated analysis throughout the development cycles revealed a consistent pattern of strong initial usability with progressive optimization in user engagement. From the first cycle, quantitative data showed excellent UIUQ scores (mean 86, SD 6), while qualitative feedback confirmed the interface was perceived as “intuitive and pleasant.” However, the convergent analysis uncovered a critical gap between this high usability perception and actual engagement metrics, with only 46% (6/13) task completion rate initially. This paradox was elucidated through user reports of forgetting to complete tasks despite appreciating the design, leading to the insight that excellent usability alone was insufficient to ensure consistent engagement. Consequently, we implemented a gamified task sequence with progressive rewards and streamlined the workflow between symptom reporting and educational content.

During the second cycle, as task completion rates rose substantially to 77% (10/13) while maintaining high UIUQ scores (mean 87, SD 6), qualitative feedback indicated that the improved task flow made it easier for users to remember subsequent actions. This convergence of metrics confirmed that structural enhancements had successfully translated the strong usability foundation into improved engagement. Building on this success, we enhanced the reminder system with contextual prompts and personalized notification timing. In the subsequent cycles 3‐4, further improvements in task completion (12/13, 92%) with stable UIUQ scores were reinforced by qualitative reports of increased enjoyment from educational games and anticipation of weekly content discoveries. This integrated understanding highlighted the importance of continuous content innovation, prompting the introduction of rotating educational mini-games and personalized knowledge recommendations based on symptom patterns to sustain long-term engagement.

### Final App Architecture

**Figure 2.**
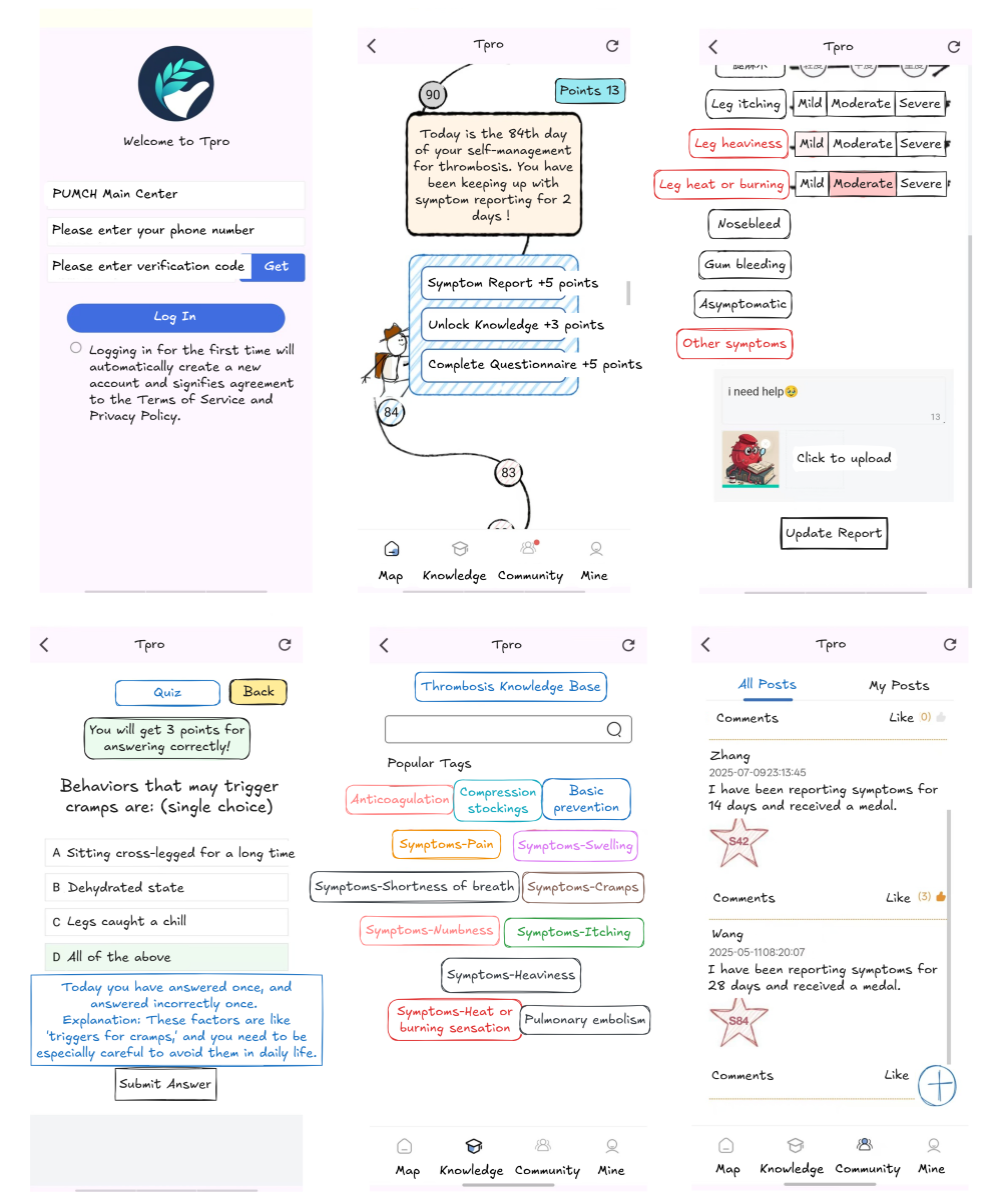
Final integrated system architecture of Tpro (English translations). The architecture comprises 4 interconnected modules developed through iterative refinement: a gamified “Exploration Map” for symptom reporting, a dynamic educational ecosystem that unlocks content based on user actions, a tiered clinician-patient communication channel with acute symptom alerts, and a professionally moderated peer support community. This design successfully integrates a high-usability interface with patient-centered functional requirements for comprehensive deep vein thrombosis (DVT) management.

The iterative development process culminated in a refined app comprising well-integrated functional modules that collectively support comprehensive DVT management. Figure 2 shows the final integrated architecture including the user onboarding, gamified reporting map, dynamic education ecosystem, and communication channels. The patient registration system evolved into a streamlined onboarding process where users complete demographic and clinical profiles after administrative verification, capturing essential information including DVT location, existing symptoms, and relevant medical history through an intuitive form-based interface.

Central to the app is the gamified symptom monitoring module. Patients are guided through a structured daily workflow, initiated by an adaptive reminder system that sends a notification at a default time of 5:00 PM, which can be customized to individual preferences. Upon entering the app, patients complete their symptom report through an “Exploration Map” interface, which transforms daily symptom reporting into an engaging, game-like activity. A carefully designed incentive mechanism awards points for consistent reporting, which accumulate toward priority scheduling for in-person follow-up appointments. For clinical support, the system features an integrated clinician-patient communication channel that enables patients to submit detailed queries with text and photographic evidence directly through the symptom reporting interface. This module operates with a sophisticated triage protocol where acute symptoms such as dyspnea trigger immediate alerts for urgent clinical response, while chronic concerns receive guaranteed feedback within a 48-hour framework.

The educational component underwent significant transformation from static information delivery to a dynamic learning ecosystem. Upon submission of each symptom report, health education content specifically matched to the symptoms reported that day is automatically unlocked and delivered through multiple formats, including illustrated materials, instructional videos, interactive quizzes, and gamified learning activities, with each educational interaction further contributing to the user’s point accumulation. At the final testing stage, the module contained 3 instructional videos, 53 illustrated materials, 292 interactive knowledge quiz items, and 45 gamified learning activities, which together constituted a content library to support rotated delivery. Based on user feedback collected during the testing phase indicating a need for continuously refreshed educational content, the clinical team will review user needs on a monthly basis and develop and add new materials accordingly. This content rotation and expansion strategy is designed to sustain long-term user engagement beyond the testing phase. For patients with a strong interest in learning, the comprehensive knowledge library remains accessible for self-directed exploration at any time, independent of the daily reporting workflow.

Complementing these core functions, the app incorporates a dedicated peer support community where patients share treatment experiences and disease management insights through text and image posts at any time. This social platform fosters mutual support through interactive features including likes and comments, while maintaining a therapeutic environment through active moderation by health care professionals.

### Administrative System Development

**Figure 3.**
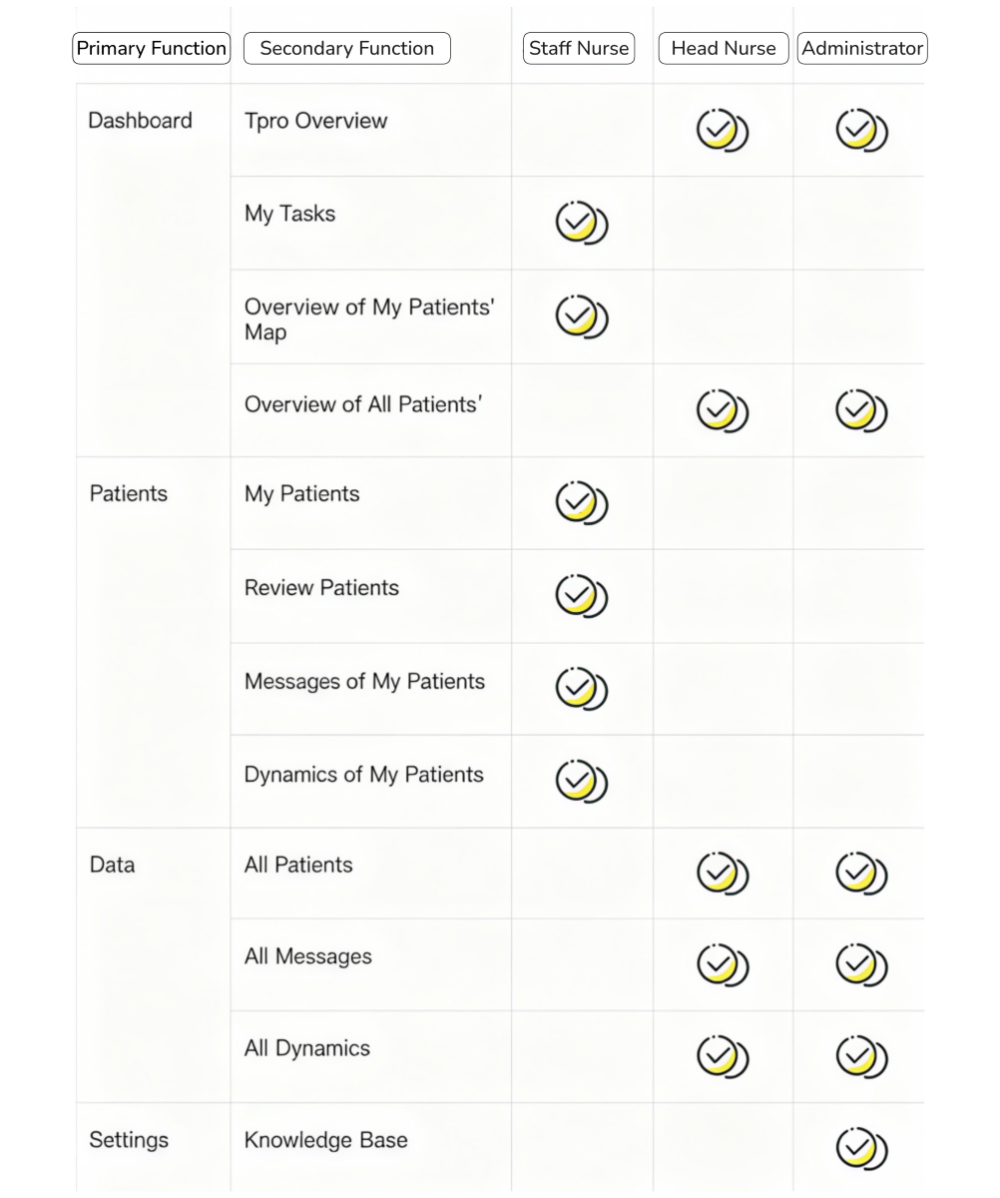
Overview of the backend administrative portal for Tpro. The portal provides role-based access controls, an analytics dashboard for tracking patient engagement, dynamic content management tools, and community moderation features. This comprehensive administrative infrastructure enables efficient patient oversight and supports institutional customization.

The backend administrative portal similarly evolved through the iterative refinement process, developing enhanced role-based permissions with granular access controls, automated flagging systems for urgent patient communications, advanced analytics dashboards for tracking patient engagement patterns, dynamic content management capabilities for educational material updates, and integrated moderation tools for community management. This comprehensive administrative infrastructure supported efficient oversight while maintaining flexibility for institutional customization. Figure 3 presents the admin portal interface with its analytics dashboard, content management, and moderation tools.

### Final Usability Outcomes

The final evaluation demonstrated outstanding usability outcomes across all metrics. The system achieved an excellent UIUQ score with a mean of 89 (SD 13) in the fourth cycle, and the task completion rate, defined as completing both symptom reporting and at least one health education activity, reached 92% (12/13), confirming high operational reliability and indicating that nearly all patients proceeded from symptom reporting to the automatically unlocked educational content, which was delivered in randomly presented formats, including illustrated materials, videos, quizzes, and games. Regarding clinician-patient communication, 12 patients generated a total of 6 inquiries across the testing period, all submitted through the symptom reporting interface. In the peer support community, 4 posts were shared across the 12 patients, while several others browsed content without actively posting. Notably, 83% (10/12) of patient participants expressed willingness to continue using Tpro and recommend it to peers, and clinical doctors and nurses consistently provided highly positive evaluations regarding the system’s clinical utility and workflow integration.

These convergent findings from multiple stakeholder perspectives substantiate that Tpro has evolved into a clinically valuable, highly usable, and widely accepted digital health intervention, positioning it as a promising tool for optimizing out-of-hospital management of DVT.

## Discussion

### Principal Findings

This study successfully demonstrates the application of an iterative convergent mixed methods design in developing Tpro, a comprehensive mHealth solution for DVT symptom management. The systematic integration of quantitative usability metrics with qualitative user feedback across multiple development cycles enabled the transformation of an initial prototype into a refined digital health intervention. The final app not only achieved excellent usability, as evidenced by a high UIUQ score (mean 89, SD 13) and task completion rate (12/13, 92%), but also received endorsement from clinical experts. More importantly, this methodology facilitated a deeper understanding of the complex interplay between technical functionality, user engagement, and clinical utility that would likely remain obscured through traditional single-method approaches.

### Comparison With Prior Work

From a design philosophy perspective, the construction of Tpro based on PRO represents an essential reshaping of the medical paradigm. Traditional clinical research has predominantly focused on objective indicators from the medical perspective. For instance, Tritschler et al’s [17] scoping review on clinical outcomes of venous thromboembolism interventions in nonpregnant adults identified recurrence, major bleeding, and mortality as the most frequently reported outcome measures. However, these metrics often fail to accurately capture patients’ real-life challenges, such as prolonged immobilization, anxiety, and depression induced by anticoagulant therapy, or the inconvenience of daily activities caused by wearing compression stockings [18,19]. In contrast, PRO directly reflects patients’ physical, psychological, and social functional experiences [20]. By collecting first-hand information through PRO, Tpro bridges the critical gap in physician-patient communication, precisely identifies patient needs, and enables targeted subsequent interventions.

The design of each core module in Tpro was meticulously informed by this PRO-driven philosophy and the specific needs uncovered in our preliminary research [11]. The symptom monitoring module uses an interactive exploration map format, while the daily reporting feature adopts a gamified approach akin to unlocking new levels. This innovative design strategically leverages game-based mechanics to transform traditionally monotonous health management tasks into engaging daily activities, fostering patient motivation through an integrated incentive system. Underlying this framework is the application of behavior change theory, which posits that patients are significantly more likely to adhere to healthy behaviors when they perceive autonomy, competence, and relatedness [14]. The multimodal health education component incorporates graphics, videos, interactive quizzes, and gamified elements to accommodate diverse learning preferences, thereby translating complex medical knowledge into easy-to-understand and easy-to-use resources [21,22].

Furthermore, Tpro actively fosters social support networks, a critical component often neglected in traditional care. The integrated clinician-patient communication channel provides a “lifeline” for acute concerns and establishes reliable, timely feedback for chronic issues, offering patients psychological security [23]. The peer support community, moderated by health care professionals, facilitates emotional resonance and the sharing of practical lived experiences, which can enhance disease-related resilience and mitigate feelings of isolation [24].

### Methodological Contributions

This study advances digital health development methodology by addressing documented limitations in conventional approaches, particularly the high abandonment rates of health apps due to poor usability [9]. Our iterative convergent mixed methods framework enabled proactive identification and resolution of usability barriers through continuous refinement based on rich, contextualized user understanding. The successful translation of user needs into design features, particularly the gamified symptom reporting and tiered communication system, aligns with evidence supporting behaviorally-informed design in digital health [9,25,26]. Beyond confirming what works, our approach systematically leverages quantitative-qualitative integration to reveal why and how features succeed across diverse users.

### Strengths

This study boasts several notable strengths. First, the adoption of an iterative convergent mixed methods design represents a rigorous and transparent methodology for mHealth development. This approach allowed for the simultaneous collection and integration of rich qualitative insights with quantitative usability metrics, enabling us to not only identify what usability issues existed but, more importantly, to understand why they occurred and how to effectively address them. Second, the development process was deeply rooted in a patient-centered philosophy, from the initial qualitative inquiry that defined the core functions to the iterative testing with end users (both patients and clinicians). This ensured that the final Tpro app was not only highly usable but also directly addressed the authentic needs and challenges faced by individuals managing DVT in their daily lives. Finally, the study delivers a fully functional and rigorously refined digital health intervention with demonstrated high usability and acceptance, which is now primed for subsequent efficacy testing, thereby providing a tangible and significant contribution to the field.

### Limitations and Challenges

Several limitations should be considered when interpreting the findings of this study. First, the generalizability of the results is constrained by the sample characteristics. All participants, including the 12 patient participants, were recruited from a single, high-resource academic medical center. Although the sample size and setting were appropriate for achieving usability saturation within the iterative convergent design framework, which was the primary aim of this development phase, they may limit the direct transferability of the findings to other contexts, such as community hospitals or populations with differing socioeconomic backgrounds, health literacy, or digital access. Second, individuals without smartphones or with significant visual or cognitive impairments were excluded, meaning accessibility barriers for these vulnerable groups remain unaddressed. Third, this version of Tpro is available only in Chinese, which necessitates thorough cultural and linguistic adaptation for broader global implementation. Fourth, engagement with the peer support community was relatively modest during the testing phase, with only 4 posts generated across the 12 patient participants. This may reflect the limited testing duration, the small user group, or the acute nature of DVT, where patients may prioritize symptom management and education over social interaction in the initial phase of self-management. The community was designed as one component of a multifaceted intervention, and the observed pattern of intermittent, need-based use may represent an appropriate level of engagement for this population. Finally, while this study demonstrates high usability and acceptance, the definitive clinical efficacy of Tpro on outcomes such as symptom burden, quality of life, and DVT recurrence requires validation through a future randomized controlled trial (RCT).

### Security Considerations

The integration of foundational data security and privacy protections, including end-to-end encryption, internal server deployment, and role-based access controls, was essential for Tpro as an mHealth tool handling sensitive patient information. These safeguards were designed to protect confidentiality, build user trust, and meet core data protection requirements. For future implementations in other settings, such controls must be adapted to local regulatory frameworks. A focus on privacy-by-design addressed a key barrier to patient adoption and supported sustainable clinical integration of digital health tools.

### Implications for Practice and Research

This study presents significant implications for both mHealth development and future research. For developers and health care organizations, the iterative convergent mixed methods approach offers a replicable framework for creating evidence-based digital health solutions that effectively balance technical sophistication with user-centered design principles. The specific design strategies developed through this process, particularly the gamification elements and tiered communication system, provide valuable reference models for other chronic disease management apps. For researchers, this methodology demonstrates how rigorous mixed methods can substantially enhance intervention development quality and impact.

Future research should prioritize a multicenter RCT to establish the clinical efficacy of Tpro. A proposed design for such a trial includes the following key components:

(1) Design and participants: a pragmatic, 2-arm, parallel-group RCT comparing Tpro plus usual care versus usual care alone. We plan to recruit approximately 200‐250 patients with confirmed DVT from multiple clinical sites, stratified by factors such as initial clot location and risk profile.

(2) Intervention: the intervention group will receive access to the Tpro app alongside standard clinical management. Usual care will follow current local guidelines without the app.

(3) Primary outcomes: the primary endpoints will focus on patient-centered outcomes, including symptom burden and health-related quality of life, assessed at 3 and 6 months postenrollment.

(4) Secondary outcomes: key secondary outcomes will include objectively confirmed DVT recurrence rates, health care use, adherence to anticoagulant therapy, and sustained engagement metrics within the app.

(5) Analysis: the primary analysis will follow the intention-to-treat principle. This definitive efficacy trial will be essential to translate the demonstrated usability of Tpro into evidence for clinical and patient-centered benefit. Beyond this RCT, subsequent research directions remain important, integrating artificial intelligence for personalized symptom management; validating the system across diverse health care systems and cultural contexts to enhance generalizability; exploring strategies to foster more active peer interaction within the community module; and conducting longitudinal studies to examine the relationship between sustained engagement and long-term health outcomes.

### Conclusions

The iterative convergent mixed methods design enabled the successful development of Tpro, a PRO-based mobile app for DVT symptom management, yielding a highly usable and clinically relevant digital health intervention. Through 4 iterative cycles integrating mixed methods research, we resolved critical usability issues while gaining profound insights into patient engagement patterns. The resulting app provides comprehensive symptom monitoring, education, communication, and peer support within an evidence-based framework, now ready for formal efficacy testing. This study validates a rigorous methodology for developing digital health solutions that effectively balance technical sophistication with human-centered design, offering significant potential to enhance out-of-hospital DVT care while establishing a replicable approach for creating meaningful solutions for patients and clinicians.

## Supplementary material

10.2196/92738 Multimedia Appendix 1 Wireframe diagram of the Tpro app (English translations). This wireframe was generated during the predevelopment phase to establish the initial structural layout of the 4 core modules. It illustrates the spatial hierarchy, modular organization, and the intended user flow among symptom monitoring, health education, clinician-patient communication, and peer support community, providing the foundational visual scaffold for subsequent iterative refinement.

10.2196/92738 Multimedia Appendix 2 Representative screenshots of the Tpro app across 4 iterative testing cycles (English translations).

10.2196/92738 Multimedia Appendix 3 Representative quotations from qualitative interviews across iterative cycles. This material supported the qualitative findings presented in the manuscript. It provided authentic participant quotes that illustrated key themes related to usability, engagement, educational content, and communication, enhancing the transparency and richness of the qualitative analysis.

10.2196/92738 Multimedia Appendix 4 Characteristics of individual participants in the iterative testing. This table provided deidentified, participant-level data for the 21 individuals involved in the study, including patients, clinical experts, and technical evaluators. It contained demographics, professional details, and specific cycle-by-cycle participation.

## Abbreviations

DVT: deep vein thrombosis
mHealth: mobile health
PRO: patient-reported outcome
RCT: randomized controlled trial
UIUQ: User Interface Usability Questionnaire
