# Supplementary figures and images for: A Mobile App (Tpro) for Symptom Management in Patients With Deep Vein Thrombosis Based on Patient-Reported Outcomes: Design and Development Using an Iterative Convergent Mixed Methods Approach
Source: JMIR Hum Factors. 2026 Jul 3;13:e92738. doi: 10.2196/92738 (PMC13331395; doi:10.2196/92738)

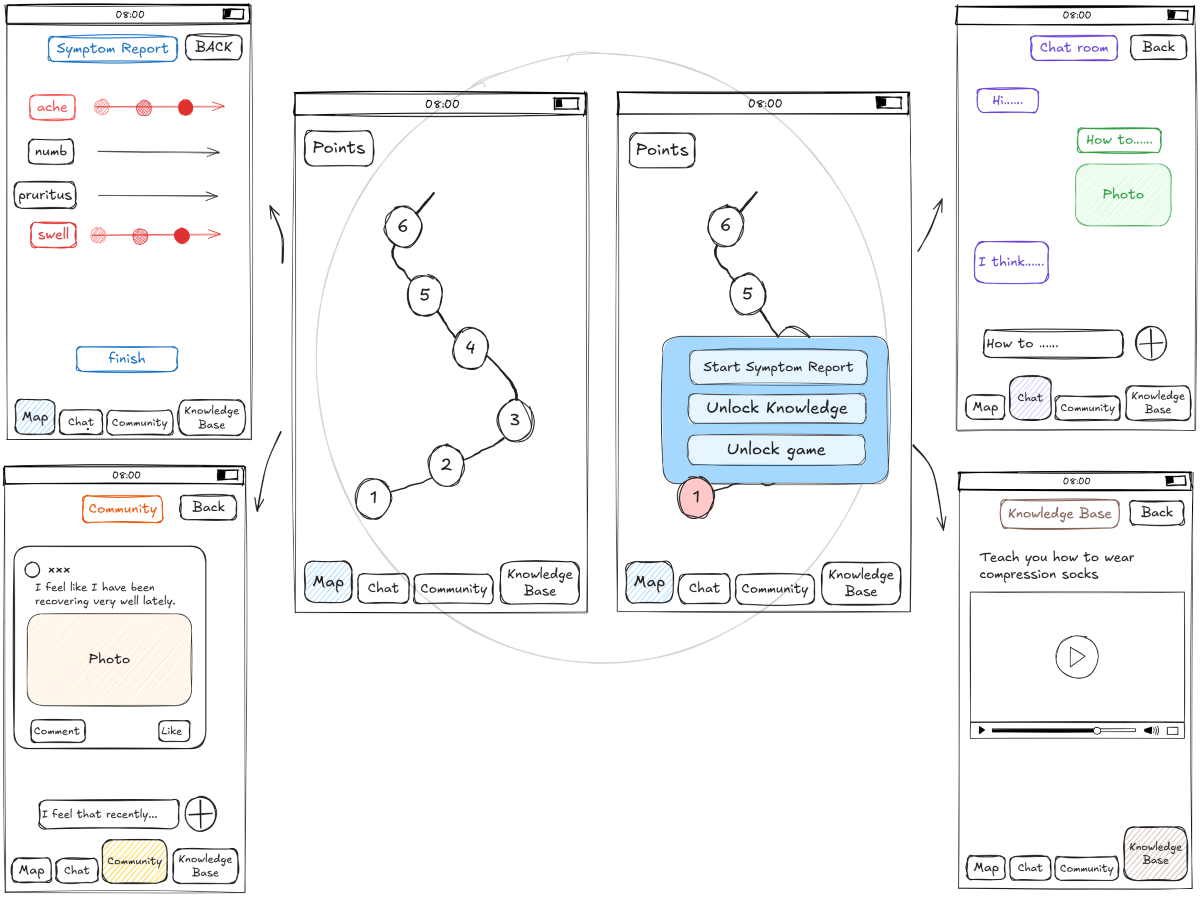

Supplement: Multimedia Appendix 1 [file humanfactors-v13-e92738-s001.png]

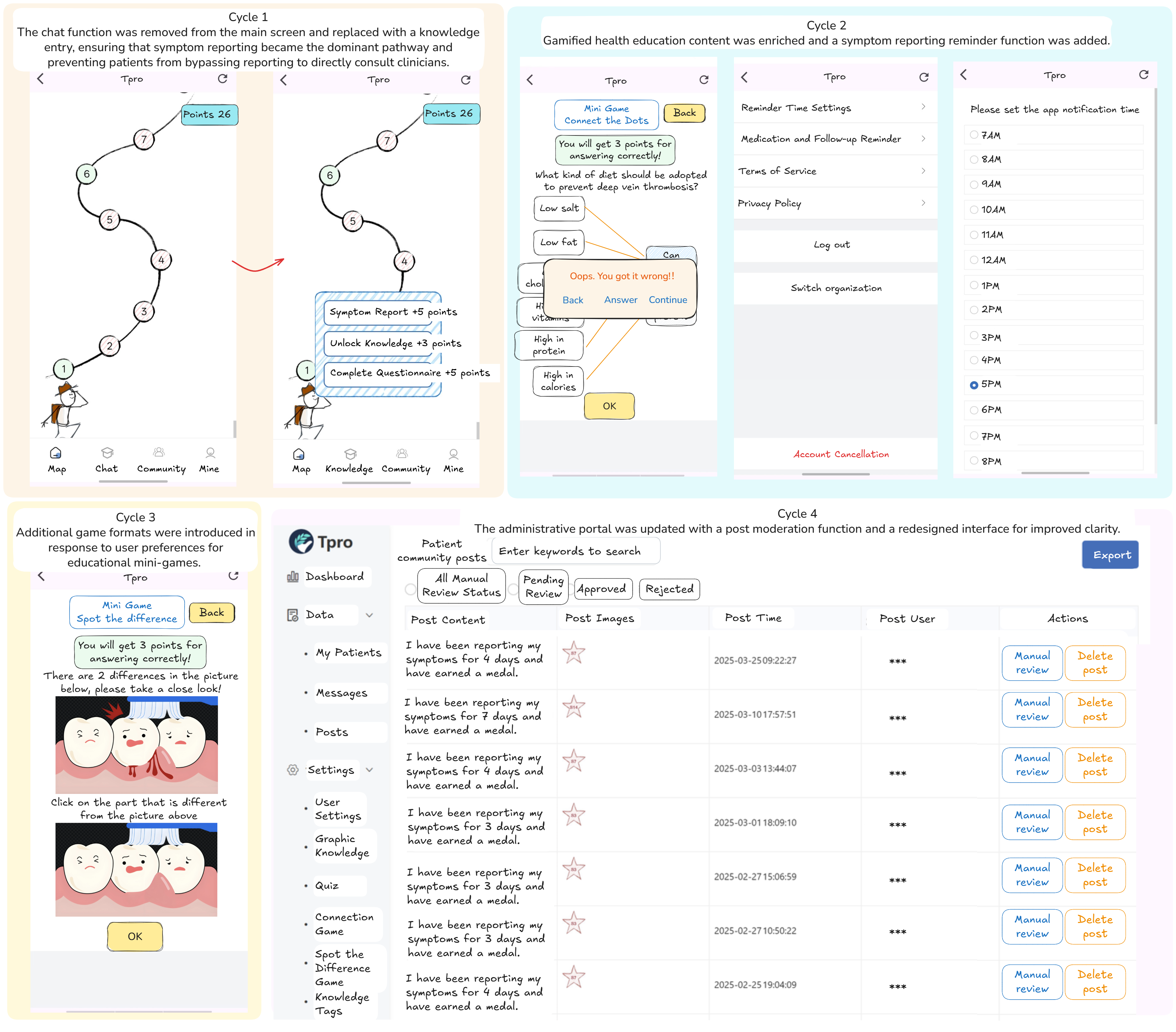

Supplement: Multimedia Appendix 2 [file humanfactors-v13-e92738-s002.png]
