# Supplementary material for: A Mobile App (Tpro) for Symptom Management in Patients With Deep Vein Thrombosis Based on Patient-Reported Outcomes: Design and Development Using an Iterative Convergent Mixed Methods Approach
Source: JMIR Hum Factors. 2026 Jul 3;13:e92738. doi: 10.2196/92738 (PMC13331395; doi:10.2196/92738)
Supplement: Multimedia Appendix 3 [file humanfactors-v13-e92738-s003.docx]

Supplementary Material. Representative quotations from qualitative interviews across iterative cycles. This material supported the qualitative findings presented in the manuscript. It provided authentic participant quotes that illustrated key themes related to usability, engagement, educational content, and communication, enhancing the transparency and richness of the qualitative analysis.

| Theme | Sub-theme | Participant ID | Representative Quotation | Cycle |
| --- | --- | --- | --- | --- |
| **Usability & Interface Design** | Distraction from Core Task | Patient 03 | *“I got distracted by the chat function on the main screen and forgot to report symptoms.”* | 1 |
|  | Clarity of System Feedback | Patient 07 | *“I wasn’t sure if my report went through-no confirmation”* | 1 |
|  | Navigation Fluency | Patient 05 | *“The navigation feels jumpy between sections”* | 1 |
| **Engagement & Motivation** | Perceived Usefulness vs. Time Burden | Patient 10 | *“The app is super convenient, but I don’t really have time to learn about thrombosis. I just check it out from time to time.”* | 1 |
|  | Gamification as Incentive | Patient 08 | *“It would be great to have more educational mini-games!”* | 1 |
|  | Content Freshness & Anticipation | Patient 02 | *“I somewhat look forward to discovering new content or small challenges each week.”* | 3 |
| **Educational Content** | Depth & Coverage of Information | Patient 11 | *“The knowledge base still has some room for improvement. There are so many topics I want to learn about!”* | 1 |
|  | Multi-format Learning Preference | Nurse 02 | *“Patients responded much better to videos and diagrams than text alone.”* | 2 |
| **Communication & Support** | Need for Reliable Clinician Feedback | Patient 09 | *“Knowing that I can ask questions to a doctor here and get a reply makes me feel more secure.”* | 4 |
|  | Value of Peer Community | Patient 12 | *“Reading about other patients’ experiences makes me feel I’m not alone in coping with this.”* | 3 |
