# Supplementary material for: A Mobile App (Tpro) for Symptom Management in Patients With Deep Vein Thrombosis Based on Patient-Reported Outcomes: Design and Development Using an Iterative Convergent Mixed Methods Approach
Source: JMIR Hum Factors. 2026 Jul 3;13:e92738. doi: 10.2196/92738 (PMC13331395; doi:10.2196/92738)
Supplement: Multimedia Appendix 4 [file humanfactors-v13-e92738-s004.docx]

Supplementary Table S1. Characteristics of individual participants in the iterative testing. This table provided de-identified, participant-level data for the 21 individuals involved in the study, including patients, clinical experts, and technical evaluators. It contained demographics, professional details, and specific cycle-by-cycle participation.

| Participants | Age (years) | Gender | Degree | DVT Location | DVT Symptoms | Years in Role | Professional Title | Cycles Completed |
| --- | --- | --- | --- | --- | --- | --- | --- | --- |
| Patient 01 | 52 | Male | High school | Left iliofemoral | Swelling, pain | - | - | 1,2 |
| Patient 02 | 68 | Female | High school | Right calf | Heaviness | - | - | 1,2 |
| Patient 03 | 41 | Male | Master | Left iliofemoral | Swelling | - | - | 1-4 |
| Patient 04 | 33 | Male | Bachelor | Left calf | Pain | - | - | 1,2 |
| Patient 05 | 59 | Male | High school | Right iliofemoral | Swelling | - | - | 1-4 |
| Patient 06 | 47 | Female | Bachelor | Bilateral calf | Swelling | - | - | 1,2,3 |
| Patient 07 | 72 | Male | Elementary school | Left femoral | Swelling, pain | - | - | 1,2 |
| Patient 08 | 25 | Male | Bachelor | Left popliteal | Pain | - | - | 1-4 |
| Patient 09 | 63 | Female | High school | Left calf | Heaviness, itching | - | - | 1-4 |
| Patient 10 | 55 | Male | High school | Right iliac | Swelling, discoloration | - | - | 1,2 |
| Patient 11 | 37 | Male | Bachelor | Left femoral | Swelling | - | - | 1,2 |
| Patient 12 | 45 | Female | High school | Bilateral calf | Pain | - | - | 1,2,3 |
| Doctor 01 | 44 | Male | Doctor | - | - | 15 (DVT Research) | Vascular Surgeon, Attending Physician | 1-4 |
| Doctor 02 | 29 | Male | Doctor | - | - | 6 (DVT Research) | Vascular Surgeon, Resident Physician | 1-4 |
| Nurse 01 | 46 | Female | Master | - | - | 8 (DVT Research) | Vascular Head Nurse | 1-4 |
| Nurse 02 | 27 | Female | Master | - | - | 5 (DVT Research) | Vascular Nurse | 1-4 |
| Nurse 03 | 31 | Female | Bachelor | - | - | 5 (DVT Research) | Vascular Nurse | 1-4 |
| Nurse 04 | 29 | Female | Master | - | - | 6 (DVT Research) | Vascular Nurse | 1-4 |
| Technical Evaluator 01 | 27 | Male | Bachelor | - | - | 5 (User Experience Research) | Software Engineer | 1-4 |
| Technical Evaluator 02 | 32 | Female | Bachelor | - | - | 7 (Development) | Human Factors Engineer | 1-4 |
| Technical Evaluator 03 | 42 | Male | Doctor | - | - | 12 (Usability) | Usability Researcher | 1-4 |
